# Low dose arsenite confers resistance to UV induced apoptosis via p53-MDM2 pathway in ketatinocytes

**DOI:** 10.1038/oncsis.2017.67

**Published:** 2017-08-07

**Authors:** Y Zhou, W Zeng, M Qi, Y Duan, J Su, S Zhao, W Zhong, M Gao, F Li, Y He, X Hu, X Xu, X Chen, C Peng, J Zhang

**Affiliations:** 1 Department of Dermatology, Xiangya Hospital, Central South University, Changsha, Hunan, China; 2 Hunan Key Laboratory of Skin Cancer and Psoriasis, Xiangya Hospital, Central South University, Changsha, Hunan, China; 3 Department of Plastic and Aesthetic Surgery, Xiangya Hospital, Central South University, Changsha, Hunan, China; 4 Department of Occupational and Environmental Health, School of Public Health, Central South University, Changsha, Hunan, China; 5 Department of Neurosurgery, Xiangya Hospital, Central South University, Changsha, Hunan, China; 6 Department of Dermatology, The First Affiliated Hospital of Dalian Medical University, Dalian, Liaoning, China; 7 Department of Dermatology, Uiversity of Pennsylvania School of Medicine, Philadelphia, PA, USA

## Abstract

Chronic arsenite and ultraviolet (UV) exposure are associated with skin tumor. To investigate the details by low concentrations of arsenite and UV induced carcinogenesis in skin, hTERT-immortalized human keratinocytes were used as a cellular model with exposure to low concentrations of sodium arsenite and UV. The effect of NaAsO_2_ on UV treatment-induced apoptosis was measured by flow cytometry and Hoechst staining. We found that the cell apoptosis induced by UV exposure was significantly attenuated after exposure to low-dose arsenite, and knockdown of p53 could block UV-induced apoptosis indicating that this phenomenon depended on p53. Interestingly, the expression of murine double minute 2 (MDM2), including its protein and transcriptional levels, was remarkably high after exposure to low-dose arsenite. Moreover, low-dose arsenite treatment dramatically decreased the MDM2 gene promoter activity, suggesting that this effect has been mediated through transcription. In addition, treatment of PD98059 reversed low-dose arsenite-induced MDM2 expression, and the inhibition of ERK2 expression could significantly block MDM2 expression as a consequence, and p53 expression automatically was increased. To validate the role of p53 in exposure to low-dose arsenite, the expression of p53 was examined by immunohistochemistry in the skin of Sprague−Dawley rats model by chronic arsenite exposure for 6 months and in patients with arsenic keratosis, and the results showed that the expression of p53 was decreased in those samples. Taken together, our results demonstrated that low-dose arsenite-induced resistance to apoptosis through p53 mediated by MDM2 in keratinocytes.

## Introduction

Human exposure to inorganic arsenic is unavoidable, because the metal is naturally present in food, soil, water and airborne particles, leading to widespread human exposure. In addition, arsenic pollution is widely prevalent in China. The inhabitants of Sanxi, Guizhou, Shanxi and Xinjiang are likely exposed to contaminated drinking water.^1^ Inorganic arsenic is considered to be a human carcinogen and a tumor-promoting agent. Exposure to arsenic may lead to chromosome instability,^2, 3^ gene mutation/amplification/suppression,^4, 5^ altered DNA repair,^6, 7^ altered transcriptional regulation,^8^ altered growth factor expression^9^ and enhanced cell proliferation. All these events are related to tumorigenesis.^8, 10, 11^ In fact, long-term exposure to inorganic arsenic has been reported to be associated with an increased risk of developing cancer in many organs, especially the skin, urinary bladder and lungs.^12, 13, 14^ An elevated incidence of skin cancers and hyperkeratosis is associated with chronic exposure to arsenic, particularly from drinking water.^15^

Tumorigenesis is a process that selects for genetic and epigenetic changes allowing evasion of antiproliferative and cell death-inducing mechanisms.^16^ Most of these genetic changes affect the tumor suppressor p53-associated pathways, which guard against DNA damage and genetic instability.^17, 18^ It has previously been demonstrated that in early or pre-tumorigenesis (before genomic instability and malignant conversion), the p53-regulated DNA damage response network is activated in an attempt to delay (cell-cycle arrest) or prevent (repair or apoptosis) mutations and cancer development.^16, 19^ Arsenic was reported to act specifically on p53 and its related proteins. In cultured HaCaT cells, arsenic exposure induced a change in the expression of p53 and the p53 regulatory protein, murine double minute 2 (MDM2).^20^ In p53-compromised cells (with either p53 dysfunction or inhibition), arsenic exposure was reported to induce centrosomal abnormality and colony formation.^21^ In p53 heterozygous knockout and wild-type C57BL/6 J mice, tumor induction by DMA (a major metabolite of ingested inorganic arsenics in most mammals), particularly of malignant lymphomas and sarcomas, was similar in treated- and control-p53(±) knockout mice.^22^

The p53 protein is one of the most important tumor suppressors. It plays a role in DNA transcription as well as in cell growth and proliferation.^23^ The p53 protein can induce cell-cycle arrest when DNA damage occurs, allowing enough time to repair the damaged DNA.^24, 25, 26, 27^ When DNA damage cannot be repaired, the p53 protein may induce apoptosis to maintain the integrity of chromosomal DNA. However, p53’s function is dependent on and related to many other genes and proteins. For example, MDM2 is one of these proteins. The human MDM2 gene is on chromosome 12q13−14, and its main biological functions include inhibiting p53’s activity by inducing its proteasomal degradation.^28^ The p53 and MDM2 proteins interact with each other to form the ‘p53/MDM2 negative feedback loop’ wherein p53 induces and activates the transcription of the MDM2 gene, then the MDM2 protein downregulates p53 by inducing its transport to the cytoplasm and its subsequent proteasomal degradation.^29, 30^ When DNA damage occurs, p53 protein expression is increased, eventually leading to activation of MDM2 transcription and expression, followed by the negative feedback loop. When p53 function is disabled, the cell genome may become unstable, and cells may undergo malignant transformation.^31^

Possible mechanisms of arsenic action have been proposed, including arsenic-induced oxidative stress, chromosomal damage, altered transcription factor levels and impaired DNA repair.^32^ However, further details of arsenic's mechanism of action still need to be elucidated. Cell models have provided valuable information on arsenic action,^33^ but studies of animal, especially human samples, are urgently needed. Research on biomarkers of arsenic-exposed human samples, such as p53, is very limited. According to the IARC, the skin shows the strongest association between chronic arsenic exposure and cancer,^34^ and dermal manifestations such as hyperpigmentation and hyperkeratosis are diagnostic of chronic arsenicosis. Arsenical hyperkeratosis appears predominantly on the palms of the hands and soles of the feet.^35^

In the present study, we chose to use human keratinocytes to study the mechanism by which exposure to arsenic can induce carcinogenesis. Cells were exposed to sodium arsenite (NaAsO_2_), which is the main form present in the environment.^36^ Cells were exposed to only low concentrations (⩽2 μM) of the compound, which is similar to the levels of total arsenic found in people having heavily contaminated blood (0.5–1.2 μM).^37, 38, 39^ The influence of sodium arsenite exposure on ultraviolet (UV)-induced cell apoptosis was studied to determine whether arsenic exposure affects response to other carcinogens. Then, the promoter activity of MDM2 was examined to further determine the mechanism underlying the effects of the arsenic. In addition, immunohistochemistry was performed to observe the expression of p53 in the skin of Sprague−Dawley (SD) rats after chronic arsenite exposure for 6 months and in patients with arsenic keratosis.

## Results

### Arsenic reduces UV-induced apoptosis in p53-dependent manner

**Figure 1:**
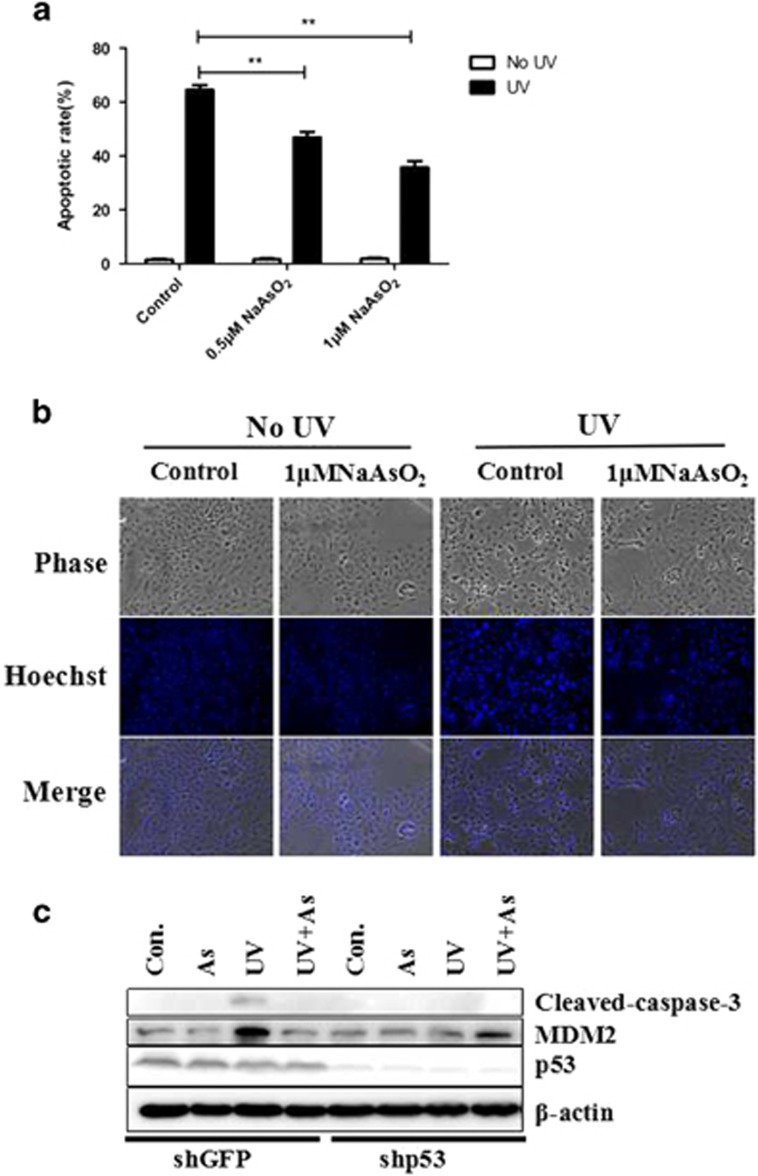
Arsenic treatment decreases cellular apoptosis induced by UV exposure. (**a**) The cellular apoptotic rate determined by flow cytometry. When cells were not exposed to UV irradiation, the rate of keratinocyte apoptosis was low, regardless of whether the cells had been pretreated with sodium arsenite. However, upon UV exposure, the apoptotic rate of control keratinocytes increased, while the cells pretreated with sodium arsenite did not experience the same extent of apoptosis. (**b**) Cell morphology detected by phase-contrast microscopy, after Hoechst 3258 staining. When cells were not exposed to UV, there was no change in morphology even in cells pretreated with sodium arsenite. When the keratinocytes were exposed to UV, pyknosis of nuclei and nucleosomes was observed. Pretreatment with sodium arsenite decreased the incidence of pyknosis. (**c**) After the knockdown of p53, the expression of apoptotic markers was detected by immunoblotting.

Apoptosis is an important way for cells to maintain genomic stability and resist tumorigenesis. Carcinogenic substances may change a cell’s propensity toward apoptosis. To determine whether this is the case with arsenic, we examined the influence of sodium arsenite on the rate of apoptosis in keratinocytes. Figure 1a shows the apoptotic rate of keratinocytes as detected by flow cytometry. Without UV exposure, the rates of apoptosis were not significantly different between the control group and the sodium arsenite groups (0.5  or 1 μmol/l). However, when the cells were pretreated with sodium arsenite and then exposed to UV, they had a decreased apoptotic rate. Compared with the control, the apoptotic ratio in the arsenic treatment groups were significantly lower (*P*<0.05) in groups treated by 0.5 μmol/l sodium arsenite (46.9%) or 1 μmol/l sodium arsenite (35.7%).

Cell morphology changes when cells are undergoing apoptosis.^40^ In addition to the changes noted by flow cytometry, we also observed changes in cell morphology. Apoptotic nuclei were observed by phase-contrast microscopy and after Hoechst 33258 staining (Figure 1b). Without UV exposure, the nuclear morphometry of keratinocytes was unchanged after treatment with sodium arsenite. After keratinocytes were exposed to UV, pyknosis of the nuclei and nucleosomes was observed. For cells pretreated with sodium arsenite, pyknosis of the nuclei and nucleosomes was less common. These results were consistent with those of the flow cytometry assay.

To further determine whether the apoptosis in response to UV under these conditions is p53-dependent, we knocked down p53 using an RNAi approach. Figure 1c shows that treatment of keratinocytes with UV-induced cleavage of caspase-3, while it is disappeared when p53 was knocked down, which demonstrates that the apoptosis in response to UV is indeed p53-dependent.

### Arsenic causes the subcellular redistribution and decreased the expression of p53.

**Figure 2:**
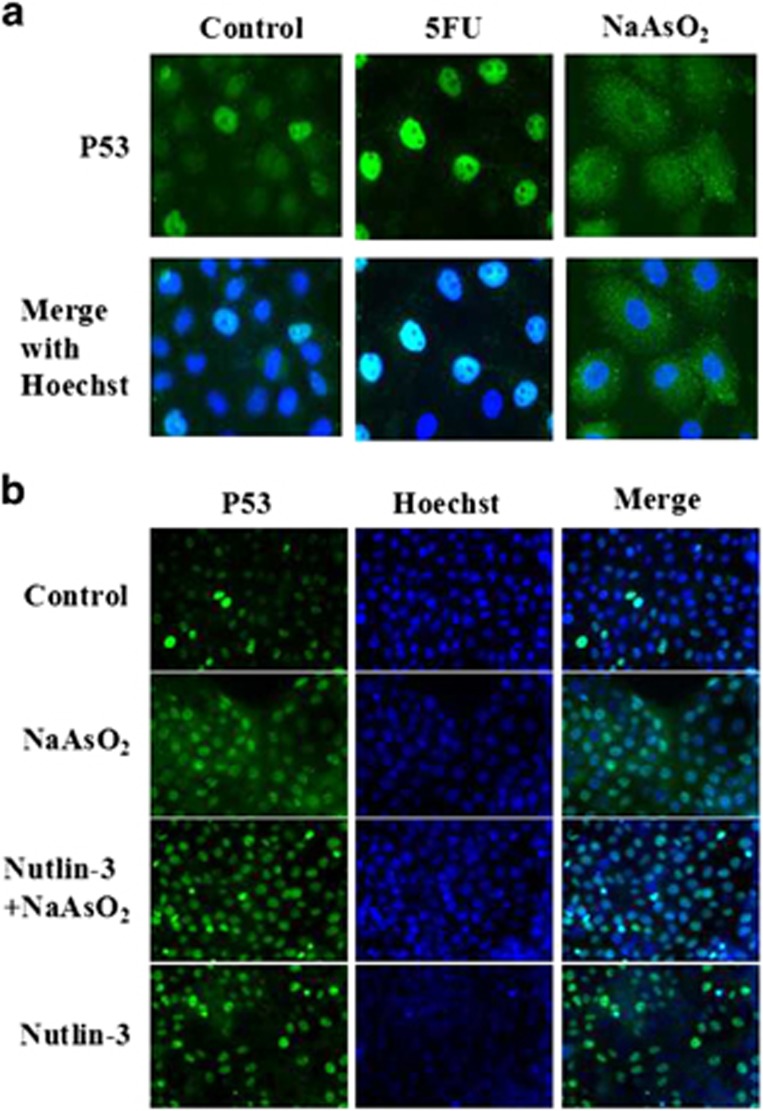
Subcellular localization of p53 detected by immnofluorescence. (**a**) Cells treated with 5-FU had localization of p53 primarily in the nucleus, while cells pretreated with sodium arsenite exhibited primarily cytoplasmic localization of p53. (**b**) After addition of Nutlin-3 to block the interaction between p53 and MDM2, p53 was mainly found in the nuclei of keratinocytes, regardless of whether the cells were treated with 5-FU or sodium arsenite.

The p53 protein is important for cell apoptosis. As we had found that sodium arsenite decreased the rate of apoptosis in response to UV exposure, we examined the expression of p53. Immunofluorescence staining demonstrated that after keratinocytes were treated with 5-Fluorouracil(5-FU), p53 was primarily localized in the nuclei of cells. On the other hand, when cells were treated with sodium arsenite, p53 was localized in the cytoplasma and the expression decreased (Figure 2a). This result was consistent with the change in the apoptotic rate, as p53’s function is dependent on its subcellular localization in the nucleus. Thus, sodium arsenite induced p53 translocation to the cytoplasm, which inhibited p53’s pro-apoptotic function.

The nuclear export of p53 is dependent on MDM2.^30^ To investigate the mechanism underlying the translocation of the protein, Nutlin-3 was employed. Nutlin-3 specifically inhibits the interaction between MDM2 and p53,^41, 42^ blocking the MDM2-mediated nuclear export of p53. For our study, cells were divided into 4 treatment groups: control, NaAsO_2_ 2 μmol/l, Nutlin-3 10 μmol/l, and NaAsO_2_ 2 μmol/l + Nutlin-3 10 μmol/l. Cells were treated for 12 h, and then immunofluorescence staining was performed. The translocation of p53 induced by sodium arsenite was inhibited by Nutlin-3. In fact, after adding Nutlin-3 to the cell culture, p53 was primarily located in the nuclei, regardless of whether the cells were treated with 5-FU or sodium arsenite (Figure 2b).

### Arsenic upregulated the expression of MDM2 in keratinocytes

**Figure 3:**
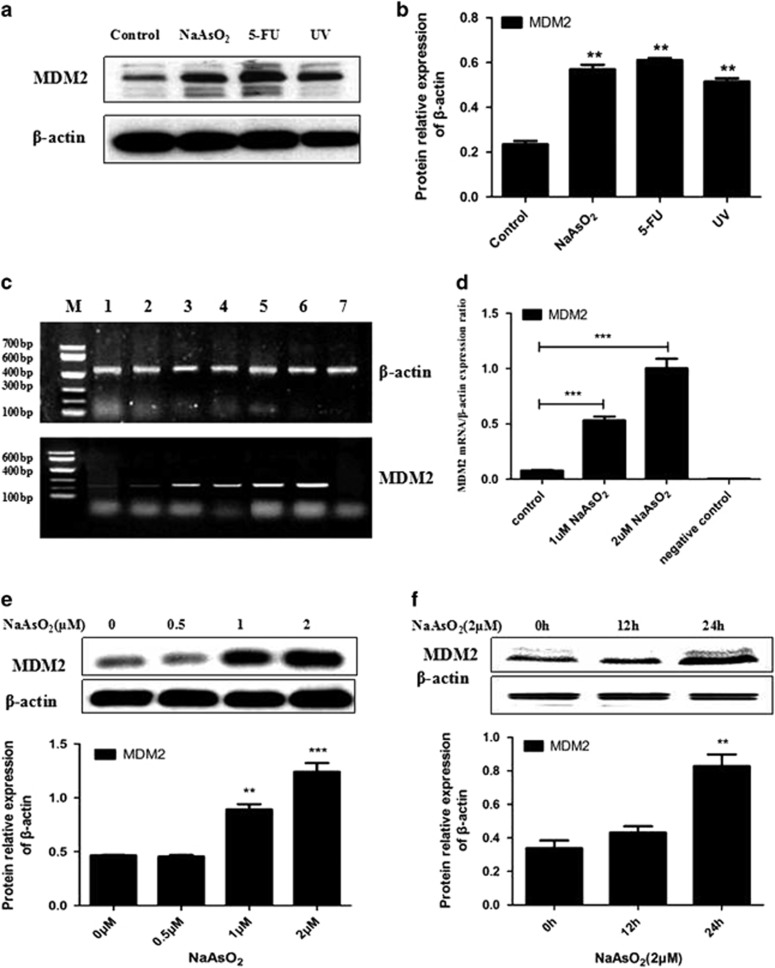
Expression of MDM2 and MDM2 mRNA. (**a**) In cells treated with sodium arsenite, the MDM2 expression was increased. When cells were treated with UV radiation or 5-FU also, the MDM2 expression was increased. (**b**) Histograms show the relative fold change of proteins (mean±s.e.m. of triplicates) using β-actin as the control of protein loading. *P<0.05 vs Control. (**c**) MDM2 mRNA expression was increased after treatment with sodium arsenite. 1–2: control, 3-4: 1 μmol/l NaAsO_2_ 24 h, 5–6: 2 μmol/l NaAsO_2_ 24 h, 7: negative control. (**d**) MDM2 mRNA relative expression ratio, ***P<0.001 vs Control. (**e**) The MDM2 expression increased following sodium arsenite exposure in a concentration-dependent manner. **P<0.01, ***P<0.001 vs control. (**f**) The MDM2 expression increased following sodium arsenite exposure in an exposure time-dependent manner. **P<0.01 vs control.

Based on these results, we concluded that MDM2 mediated p53’s subcellular relocation when keratinocytes were treated with sodium arsenite. We next wanted to examine the effect of sodium arsenite on the expression of MDM2. Figure 3 shows the effect of sodium arsenite on the level of MDM2 mRNA and protein. After the cells were treated with sodium arsenite, the expression of the MDM2 protein was upregulated, similar to the effects of UV and 5-FU (Figures 3a and b). Furthermore, the overexpression of MDM2 protein increased with the concentration of sodium arsenite and the time duration (Figures 3e and f). The expression of *MDM2* mRNA was also upregulated after keratinocytes were treated with sodium arsenite (Figures 3c and d). As with the protein, the overexpression of *MDM2* mRNA increased with the concentration of sodium arsenite.

### Arsenic affects MDM2 promoter activity and transcription via the MAPK/ERK pathway

**Figure 4:**
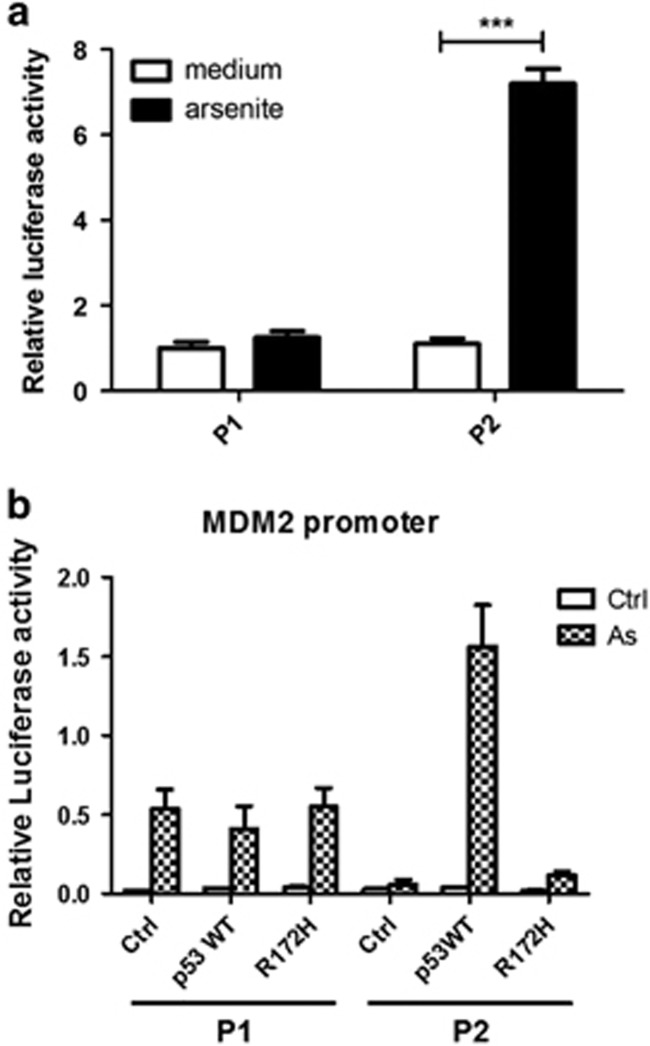
MDM2 promoter−luciferase activity in transfected cells. (**a**) The luciferase activity of untreated cells transfected with pGL_3_-MDM2 P1/P2 was four times that of cells transfected with the pGL_3_-control (*P*<0.05). After treatment with sodium arsenite, the luciferase activity of cells transfected with pGL_3_-control or pGL_3_-MDM2 P1 was not significantly changed (*P*>0.05); however, the luciferase activity of cells transfected with pGL_3_-MDM2 P2 was seven times that of cells transfected with pGL_3_-control (*P*<0.05). (**b**) Effects of knockdown of p53 on NaAsO_2_-induced MDM2 promoter activity. Keratinocytes expressing p53-WT and p53 R172H treated with 2 μmol/l NaAsO_2_ for 48 h. Each data point represents the average of three independent biological replicates±s.e.m.

One reason for the MDM2 overexpression may be an increase in the MDM2 promoter activity. The *MDM2* gene includes two promoters, P1 and P2.^41, 42^ After recombination of pGL_3_ with MDM2 promoter P1/P2, the vector was transfected into keratinocytes. The luciferase activity represents the activity of the MDM2 promoters P1 and P2 (Figure 4a). Without sodium arsenite treatment, the luciferase activity of cells transfected with pGL_3_-MDM2 P1/P2 was four times that of cells transfected with pGL_3_-control (*P*<0.05). After treatment with sodium arsenite, the luciferase activity of cells transfected with pGL_3_-control or pGL_3_-MDM2 P1 did not change significantly (*P*>0.05); however, the luciferase activity of cells transfected with pGL_3_-MDM2 P2 was seven times that of cells transfected with pGL_3_-control (*P*<0.05). The enhanced luciferase activity of cells transfected with pGL_3_-MDM2 P2 indicated that activity of MDM2 promoter P2 was enhanced by the sodium arsenite treatment.

To investigate the effects of knockdown of p53 on arsenite-induced MDM2 promoter activity, the MDM2 luciferase reporter gene and p53 plasmid were cotransfected with keratinocytes, then the cells were treated with NaAsO_2_ (2 μmol/l) and its effect on MDM2 promoter activity were detected. The activity of NaAsO_2_-induced P2 promoter of MDM2 decreased after the knockdown of p53 (Figure 4b), which suggests that the effects on MDM2 expression are p53-dependent.

**Figure 5:**
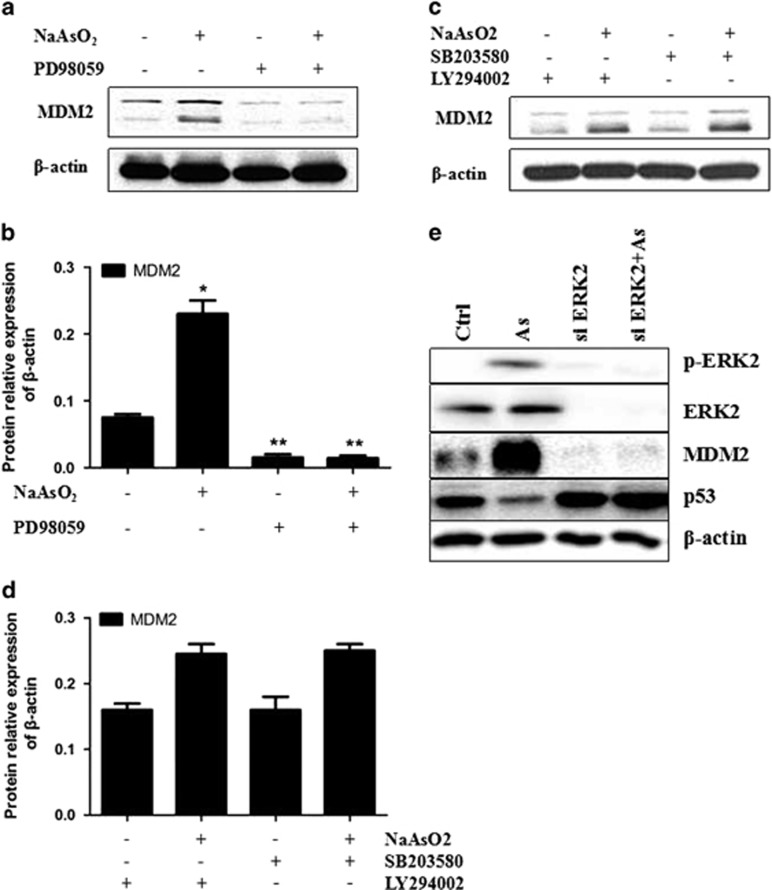
The expression of MDM2 after pretreatment with specific signal pathway inhibitors PD98059 (MAPK/ERK pathway inhibitor), SB203580 (p38 MAPK pathway inhibitor) and LY294002 (PI3K/AKT pathway inhibitor). (**a**, **b**) PD98059 inhibited the overexpression of MDM2 induced by sodium arsenite. (**c**, **d**) SB203580 and LY294002 did not have any significant effect. (**e**) Effects of ERK1/2 siRNA on arsenite-induced expression of p53 and MDM2. Immunoblot analysis of hTERT-immortalized human keratinocytes transfected with ERK1/2 siRNA or control siRNA for 48 h, followed by treatment with 2 μmol/l arsenite for 24 h.

To determine how MDM2 promoter activity was being enhanced, we made use of specific pathway inhibitors (PD98059—MAPK/ERK pathway inhibitor, SB203580—p38 MAPK pathway inhibitor and LY294002—PI3K/AKT pathway inhibitor) to block the signal pathways known to regulate MDM2, then checked the expression of MDM2, hoping to find out through which pathway the MDM2 promoter activity and expression were enhanced. The expression of MDM2 after pretreatment with specific signal pathway inhibitors was measured by Western blot. PD98059 inhibited the overexpression of MDM2 induced by sodium arsenite, whereas SB203580 and LY294002 did not have any significant effect (Figures 5a−d). These results indicate that sodium arsenite upregulated MDM2 promoter activity through the MAPK/ERK pathway.

### Arsenite-induced expression of MDM2 and p53 could be reversed by ERK1/2 knockdown

To demonstrate the effects of ERK1/2 knockdown on NaAsO_2_-induced expression of p53 and MDM2, we performed siRNA-mediated knockdown of ERK1/2. We found that arsenite-mediated enhancement of MDM2 expression and detraction of p53 expression was blocked by ERK1/2-specific siRNA but not by a negative control siRNA (Figure 5e). The observation demonstrates that sodium arsenite upregulated MDM2 expression and downregulated p53 expression through the MAPK/ERK pathway.

### Pathological changes in the skin of rats after arsenite exposure and in patients with arsenic keratosis

**Figure 6:**
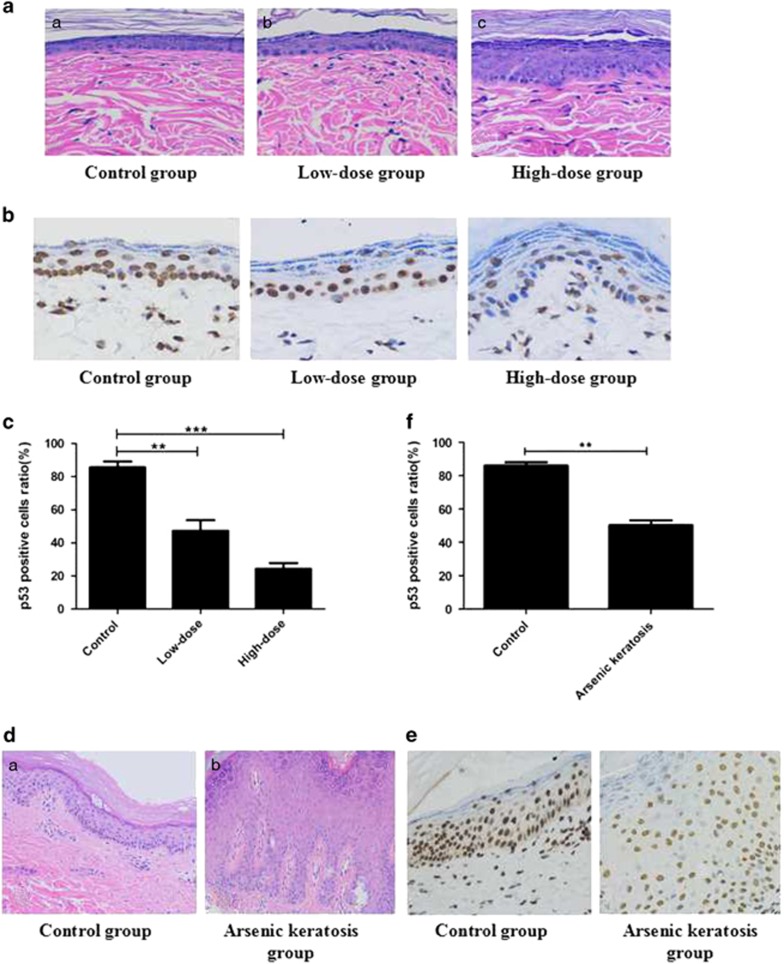
Pathological changes and immunohistochemical staining of the skin of rats after arsenite exposure in patients with arsenic keratosis.(**a**) Skin structure in each group of rats exposed to NaAsO2 for 6 months. (a) Control group. (b) Low-dose group. (c) High-dose group. hematoxylin and eosin staining × 200. (**b**, **c**) The expression of p53 (Original magnification × 400) was prominently reduced after arsenite exposure (data represented as mean±s.d., *n*=10, *P<0.05,**P<0.01 vs control). (**d**) Skin structure in the palm of hand skin lesions of patients with arsenic keratosis. (a) Control group. (b) Arsenic keratosis group. hematoxylin and eosin staining × 200. (**e**, **f**) In patients with arsenic keratosis, the expression of p53 was shown as nuclear staining of the finger extension side skin and was decreased comparing with the control (data represented as mean±s.d., *n*=10, *P<0.05,**P<0.01 vs control).

Changes in the skin of rats were observed by hematoxylin and eosin staining. The structure of the skin of rats in the control group was basic, normal, thickness of the epidermis was uniform and the epidermal cells were arranged neatly. But the skin of rats exposed to low-dose arsenite had mild keratosis. High-dose group showed severe skin keratosis and uneven skin thickness, caused by a part of the nucleus to shrinking, dissolving or disappearing (Figure 6a). In comparison, the control group showed skin structure that was basic normal , and pathological examination of the palm of hand skin lesions of patients with arsenic keratosis showed severe hyperkeratosis, epidermal hyperplasia, acupoint hypertrophy, irregular epidermis extension, accompanied by mild keratinocyte dysplasia and dermal chronic inflammatory cell infiltration(Figure 6d).

### NaAsO_2_ downregulated the p53 expression in the skin of high-dose SD rats group and in patients with arsenic keratosis

The expression of p53 via immunohistochemical (original magnification × 200) staining of the skin from the three groups of rats and in patients with arsenic keratosis was examined next. The results revealed that the expression of p53 in arsenic-exposed groups was lower than that in the control group. Moreover, expression of p53 in the high-dose group was the lowest and extremely lower than that in the control group (*P*<0.01), and significant difference also existed between the low-dose group and the control group (*P*<0.05) (Figures 6b and c). Similar results were observed with lesions in the palm of hand skin of patients with arsenic keratosis (Figures 6e and f). The expression of p53 in the skin of arsenic keratosis was decreased when compared with the control.

## Discussion

Carcinogenesis is closely related to cell apoptosis. Under normal conditions, apoptosis serves a protective function against harm from the environment by clearing mutant cells or cells with DNA damage, and preventing their transformation into malignant cells. When apoptosis is inhibited or reduced, the damaged or mutant cells may undergo proliferation, allowing for accumulation of mutant or damage cells, potentially resulting in carcinogenesis. In the present study, pretreatment with a low concentration of sodium arsenite decreased the rate of apoptosis in keratinocytes after UV exposure. UV exposure is the main environmental cause of skin cancer.^43, 44^ UV causes DNA damage, inducing activation of ataxia telangiectasia-related kinase and/or ataxia telangiectasia mutated kinase; then p53 is activated through phosphorylation of ser-15 and ser-37.^45^ Activated p53 then upregulates the expression of Bax. After Bax is transferred to the mitochondrial outer membrane, the mitochondria release pro-apopototic proteins such as cytochrome c, AIF and Smac/DIABLO, and the cells undergo the process of apoptosis.^46^

By immunofluorescence staining, we found that p53 was translocated from the nucleus to the cytoplasm and the expression was decreased. In earlier studies, the p53 protein levels in human keratinocytes decreased in a time- and dose-dependent manner after exposure to 0–1 μM arsenite.^20^ In other cell lines (HeLa, Jurkat and a lymphoblast cell line transformed with EBV), arsenite exposure (1–50 μM) led to an increase in the p53 protein level.^47^ However, this effect was not observed in C-33A cells.

Using immunofluorescence staining, we found that the subcellular location of p53 had changed from the nucleus to the cytoplasm following arsenite exposure. This could explain why p53 was not activated in these cells, as p53 function is dependent on its subcellular location. In the cytoplasm, p53’s function would be inhibited, and p53 would be subject to proteasomal degradation.^29^ The change in the subcellular localization of p53 may also explain the difference between our current results and those of an earlier study,^20^ longer (14 day) exposure to arsenite is likely to have caused more p53 accumulation in the cytoplasm, and, after p53 was degraded, the absolute p53 protein level would have been lower than that after the short treatment (24 h).

As we observed that p53 was mainly located in the cytoplasm after sodium arsenite exposure, and as p53’s nuclear export is dependent on MDM2,^30^ we examined the mRNA and protein expression of MDM2. Many researchers have investigated the relationship between p53 and MDM2. Almost all of the known activities of p53 are reduced by MDM2, either by inhibition of its function or by increasing the rate of degradation of the p53 protein. The levels of MDM2 are regulated by p53 forming a negative feedback loop controlling p53’s activity.^48^ Overexpression of MDM2 also inhibits p53-mediated transactivation.^49^ In the present study, both the mRNA and protein expression levels of MDM2 were increased in a dose- and time-dependent manner after treatment with sodium arsenite. These results were consistent with an earlier study.^20^ The MDM2 gene is overexpressed in several human tumors.^50^ At least 3 889 tumor tissue samples have been examined for MDM2 amplification, and the overall frequency of MDM2 amplification in the tumor samples is 7%.^42^

Our research results indicate that the mechanism by which sodium arsenite can promote malignant transformation is by disrupting the p53-MDM2 negative feedback loop. By constructing a recombinant plasmid with luciferase as a reporter gene, we found that the activity of the MDM2 P2 promoter was increased seven times. This was consistent with the amplification of MDM2 in human cancers.^51^ We also investigated the possible signaling pathway responsible for the enhanced P2 promoter activity. The MAPK/ERK pathway inhibitor PD98059 inhibited the overexpression of MDM2 induced by sodium arsenite. This indicates that sodium arsenite upregulated the MDM2 promoter activity through the MAPK/ERK pathway. Furthermore, immunohistochemistry was performed to observe the expression of p53 in the skin of SD rats after chronic arsenite exposure for 6 months and in patients with arsenic keratosis, and we found that the expression of p53 was decreased after arsenite exposure.

In summary, our research demonstrated that low-dose arsenite conferred resistance to apoptosis induced by UV dependent on p53 mediated via MDM2 in keratinocytes. These findings may be helpful to understand the induction of carcinogenesis by arsenite exposure, and they may provide targets for intervention to prevent or decrease the damage done to the human population exposed to arsenite pollution.

## Materials and methods

### Chemicals and reagents

Sodium arsenite (NaAsO_2_), insulin, 5-FU, Nutlin-3, Hoechst 33258, specific signal pathway inhibitors (PD98059, SB203580, LY294002), trypsin and propidium iodide (PI) were purchased from Sigma Aldrich Co (St. Louis, MO). High-glucose Dulbecco's Modified Eagle Medium (DMEM, liquid) and fetal bovine serum (FBS) were purchased from Hyclone Co. (Logan, UT, USA). Mouse anti-human MDM2 monoclonal antibody, mouse anti-human p53 monoclonal antibody, mouse anti-human β-actin monoclonal antibody, fluorescein isothiocyanate (FITC)-labeled goat anti-mouse secondary antibody, horseradish peroxidase (HRP)-labeled rabbit anti-mouse IgG and DNA marker were purchased from Santa Cruz Biotechnology (Santa Cruz, CA, USA). Cell lysis buffer for Western blotting, protein sample buffer for SDS-PAGE and substrate of HRP-ECL Chemiluminescence were purchased from Pierce Co., Huntsville, AL, USA. The reverse transcription kit, T4 DNA ligase, pGEM-T, pGL3-control, pGL3-enhancer and pCMV-β were purchased from Promega Co., Madison, WI, USA. Trizol, LipofectAMINE2000 and primers for PCR were purchased from Invitrogen Co., Huntsville, AL, USA. The plasmid Miniprep Kit and PCR product recollection kit were purchased from Omega Co., Logan, UT, USA. Taq DNA polymerase and restriction enzymes (*Xho*l, *Hind*III) were purchased from NEB Co. (Ipswich, MA, USA). DNA segment recollection kit was purchased from MBI Co., Huntsville, AL, USA.

### Cell and culture

hTERT-immortalized human keratinocytes were kindly donated by Professor Yuan Zhimin (Department of Cancer Cell Biology, Harvard School of Public Health, Boston, MA, USA). Cells were cultured in high-glucose DMEM supplemented with 10% FBS, 100 units/ml penicillin and 100 mg/ml streptomycin in an atmosphere of 5% CO_2_ and 100% humidity at 37 °C.

### Animals and treatment

Thirty healthy male SD rats aged 6 weeks weighing between 130 and 160 g were purchased from Silaike Company (Changsha, China). The animals' room was on a 12 h light/dark cycle, and the temperature was maintained at 22±2 °C and humidity of 50±15%. Rats were acclimatized to the animal facility for a week on standard rat chow and distilled water before treatment.Then they were randomly divided into three groups (*n*=10 each); three groups were exposed to NaAsO_2_ for 6 months. The three groups consisted of control group, low-grade group and high-grade group. NaAsO_2_ was administered in drinking water at concentrations of 0, 2.5 and 10 mg/kg.The dose of NaAsO_2_ was determined on the basis of LD50 values in the rats.^52^ During the whole experimental period, animals had free access to food and water. At the end of the treatment regimen, rats from each group were killed under 10% chloral hydrate anesthesia. The skin (0.5 × 0.5 cm) was fixed in 4% paraformaldehyde for histopathology. All experimental procedures complied with international guidelines and the legislation of China.

### Clinical sample collection

This study was approved by the local ethics committee. Both, patients with arsenic keratosis and healthy subject or their legal guardians provided written and informed consent. Individuals were recruited for sampling and were all checked by a physician to ensure the presence or absence of skin disease. Arsenic keratosis palm samples (lesional and adjacent non-lesional samples from the same palm) were collected from the patients who confirmed and were treated at our hospital. Non-exposed samples (control) were obtained from individuals with no prior history of arsenic exposure or any skin disease. The collected samples were fixed in 4% paraformaldehyde for histopathology.

### Cell apoptosis assay

The cell apoptosis rate was analyzed by flow cytometry. Before flow cytometric analysis, keratinocytes were divided into three groups. One was a control group, one group was incubated with 0.5 μmol/l NaAsO_2_ for 24 h and the other was incubated with 1.0  μmol/l NaAsO_2_ for 24 h. Then, the cells in all groups were exposed to 40 mJ/cm^2^ UV and grown for another 24 h. After UV exposure, the cells were lysed using trypsin and resuspended. Cells were washed with PBS (4 °C) twice, centrifuged (1000 r.p.m., 5 min), fixed with alcohol (70%, 4 °C) and incubated overnight. Before analysis, cells were washed with PBS (4 °C) twice again, then incubated in 50 μl staining solution (250 μg/ml propidium iodide and 5 μg/ml RNaseA) for 30 min. For cytometric analysis, the exciting wavelength was 480 nm and the emission wavelength was 630 nm. Cells that exhibited an apopotic peak at the G1/G0 phase were considered to be apoptotic cells. The apoptotic cell rate was analyzed using the Cell Quest Software Program (BioDiscovery, Hawthorne, CA, USA).

In addition to cytometric analysis, cells were stained with Hoechst 33258 to check for apoptotic nuclei. Cells were grouped, treated with NaAsO_2_ and exposed to UV as described. Then cells were washed twice with PBS and fixed with a 40 g/l paraformaldehyde solution for 10 min. After washing the cells with water three times, cells were incubated with 5 mg/l Hoechst solution for 10 min. Cells were then washed with water again three times, and then dried in air at room temperature. Cells were observed under a fluorescence microscope at an excitation wavelength of 340 nm.

### Western blot analysis

Before western blotting, keratinocytes were divided into six treatment groups: control (no treatment), 0.5 μmol/l NaAsO_2_, 1 μmol/l NaAsO_2_, 2 μmol/l NaAsO_2_, 5-FU and UV. When cells were 80% confluent, the various groups were treated with their respective compounds for 24 h. Cells in the control group were incubated in serum-free DMEM for 24 h. Protein extracts for Western analysis were obtained according to the protocol described previously.^53^ The protein concentration was determined using the Bradford assay. The protein was separated by SDS-PAGE electrophoresis, electrotransferred to nitrocellulose filters, and then the filters were incubated in blocking solution at 4 °C for 2 h to block nonspecific antibody binding. Subsequently, the filters were incubated in primary antibody solution for 1 h at room temperature. After that, the filters were soaked and washed in TTBS three times. Then filters were incubated in secondary antibody solution for 1 h at room temperature. The filters were soaked and washed in TBST for an additional three times. The filters were developed by the HRP-ECL method. Experiments were carried out in triplicates.

### Immunofluorescence staining

After the medium was discarded, cells were fixed in 4% paraformaldehyde solution (0.01 mol/l PBS, pH 7.2) for 15 min at room temperature. Then cells were washed wtih 0.01 mol/l PBS (pH 7.2) three times and incubated in 10% goat serum solution (0.02 mol/l PBS, pH 7.2)) at room temperature for 30 min to block nonspecific antibody binding. Subsequently, cells were incubated in primary antibody solution (mouse anti-human p53 monoclonal antibody, 1:200) at 4 °C overnight. After that, cells were soaked and washed in 0.02 mol/l PBS (pH 7.2) three times. Cells were then incubated in secondary antibody solution (FITC-labeled goat anti-mouse) for 2 h at room temperature in the dark. Cells were washed with 0.02 mol/L PBS (pH 7.2) three times and stained with 5 μg/ml Hoechst-22358 for 30 min at room temperature in the dark. Subsequently, cells were washed and the slides were mounted. Cells were observed under fluorescence microscopy. To determine the subcellular location of p53, the excitation wavelength was 495nm and the emission wavelength was 520 nm. For nuclear observation, the excitation wavelength was 346 nm and the emission wavelength was 460 nm. The control groups were not incubated with the primary antibodies, but the other procedures were the same.

### Determination of mRNA expression

Cells were treated with 1 μmol/l or 2 μmol/l Na_2_AsO_2_ for 24 h.Total cellular RNA was extracted using Trizol, according to the manufacturer’s instructions. The concentration/purity of total RNA was measured using a spectrophotometer (A260/A280), and the integrity of the RNA was verified by 1% agarose gel electrophoresis. Complementary DNA was synthesized using the purified RNA as the reverse transcription template. For MDM2, the product was 228 bp. The upstream primer was 5′-TAGACCTGTGGGCACGGACGCA-3′ and the downstream primer was 5′-GTCTCTTGTTCCGAAGCTGG-3′. For β-actin, the product was 500 bp. The upstream primer was 5′-GTGGGGCGCCCCAGGCACCA-3′ and the downstream primer was 5′-CTCCTTAATGTCACG CACGATTT-3′. The reaction was carried out as follows: 94 °C for 2 min, 30 cycles (95 °C 30 s, 56 °C 30 s, 72 °C 30 s), and 72 °C for 5 min. The products were analyzed by 1% agarose gel electrophoresis. The relative gray-scale ratio was considered to reflect the expression level of the mRNA: relative gray-scale ratio=gray-scale of target gene/gray scale of β-actin. The gray scale was determined using Image Tool 3.0, BioDiscovery, Hawthorne, CA, USA.

### Preparation of pGL3-MDM2 promoter P1/P2

Luciferase and β-galactosidase expression vectors under the control of the P1 or P2 MDM2 promoters were prepared as described previously.^54^ Cells were cultured normally, then their genomic DNA was extracted and analyzed for purity and concentration by agarose gel electrophoresis and spectrophotometry. The primers for promoters P1 and P2 were designed according to the reported human MDM2 sequence,^55, 56^ and *Xho*I and *Hind*III restriction sites were introduced upstream or downstream of the primers. For the P1 promoter, the upstream primer was 5′-CTGGGGAGTCTCGAGGGACC-3′ and the downstream primer was 5′-CTGCCTCAAGCTTCTTACGT-3′. For the P2 promoter, the upstream primer was 5′-CCTGTGTCTCGAGAAGATGG-3′ and the downstream primer was 5′-CTAAGCTTCGAGTCTCCTGT-3′. After amplification by PCR and purification by agarose electrophoresis, target DNA segments (P1 or P2) were recovered. Then the target DNA segments were linked with the pGEM-T vector. The ratio of the target DNA segments/vector was 3:1. The linkage reaction was performed at 16 °C overnight. The products of the linkage reaction were transferred into competent DH5α cells (Clontech, Mountain View, CA, USA). After the bacteria were cultured in LB agarose medium and their DNA were analyzed by cleavage electrophoresis, the positive clones were selected for amplification and preparation of a small amount of the target plasmid DNA. The plasmid DNA, pGEM-MDM2 promoter, was digested by *Xho*I and *Hind*III. The restriction fragments were analyzed by 1% agarose gel electrophoresis. The sequence of the restriction fragments was also measured by Invitrogen Co. to ensure that the gene sequence of the recombinant plasmid pGEM-MDM2 promoter was correct. Plasmid pGL3-enhancer was also digested by *Xho*I and *Hind*III and formed cohesive ends. Then, the fragment pGEM-MDM2, which included P1 or P2 was linked with the pGL3-enhancer linear fragment, and the respective products were called pGL_3_-MDM2 P1 or pGL_3_-MDM2 P2.

### Luciferase activity assay

Cells were respectively transfected with PCMV-β and pGL_3_-MDM2 P1/P2, or pGL_3_-control. Then cells were divided into two groups: one was treated with 2 μmol/l NaAsO_2_ for 24 h, the other was treated with normal culture medium. Cells were lysed to measure the luciferase activity. The ratio of the activity of pGL_3_-MDM2 P1/P2 vs that of the pGL_3_-control was considered as the luciferase activity.

### RNA interference

Stable cell lines expressing p53 shRNA constructs were maintained in 2 mg/ml puromycin. p53 shRNA plasmids were a kind gift from Professor Yuan Zhimin (Department of Cancer Cell Biology, Harvard School of Public Health, Boston, MA, USA). RNAi sequences of shRNA clones used in study are as follows:

P53_KD_F

5′-CCGGGACTCCAGTGGTAATCTACTTCAAGAGAGTAGATTACCACTGGAGTCTTTTT-3′

P53_KD_R

5′-AATTAAAAAGACTCCAGTGGTAATCTACTCTCTTGAAGTAGATTACCACTGGAGTC-3′

GFP_KD_F

5′-CCGGAAGCTGACCCTGAAGTTCATCCTCGAGGATGAACTTCAGGGTCAGCTTTTTTT-3′

GFP_KD_R

5′-AATTAAAAAAAGCTGACCCTGAAGTTCATCCTCGAGGATGAACTTCAGGGTCAGCTT-3′

For siRNA-mediated knockdown of ERK, p42 MAP Kinase (Erk2) Antibody(9108) and Phospho-p44/42 MAPK (Erk1) (Tyr204)/(Erk2) (Tyr187) (D1H6G) (5726) were purchased from Cell Signaling Technology (Danvers, MA, USA). MISSION esiRNA esiRNA human MAPK1 (esiRNA1)(EHU044171) was purchased from Sigma Life Science and Biochemicals. Cells were transfected with each respective siRNA using Lipofectamine RNAiMAX Transfection Reagent (Thermo Fisher Scientific, Huntsville, AL, USA) according to the manufacturer's protocol.

### Signaling pathway assay

Cells were regularly cultured to log phase growth, then cultured in serum-free medium for another 24 h. KC cells were pretreated with specific pathway inhibitors before incubation with sodium arsenite: PD98059 (MAPK/ERK pathway inhibitor), SB203580 (p38 MAPK pathway inhibitor) and LY294002 (PI3K/AKT pathway inhibitor). Signaling pathway inhibitors PD98059, SB203580 and LY294002 were added to cell-culture media, and cells were incubated with the inhibitors for 2 h to block the specific pathway. Then, cells were divided into treatment (2 μmol/l NaAsO_2_ for 24 h) and control (normal culture medium) groups. After that, cells were lysed, and Western blotting was performed to measure the expression of MDM2 and p53.

### Hematoxylin and eosin staining

The tissues taken from 4% paraformaldehyde were rinsed with running tap water, then the tissues were dehydrated in a gradient of high-percentage ethanol (70−100%). Then tissues were paraffin-embedded and sliced into serial coronal sections (3 μm thickness) with microtome (2245, Leica,Germany). The sections were deparaffinized in xylene and rehydrated in a gradient of high-percentage ethanol (70−100%), and then washed in distilled water for 5 min. They were stained with hematein for 10 min, then rinsed with running tap water. They were then stained with eosin for 1 min and subsequently immersed in 1% acid alcohol for 10 s. After being dehydrated in alcohol and cleared in xylene, neutral resin was used to mount. To evaluate the histopathological change of skin, sections were examined under a light microscope (Olympus, Japan).

### Immunohistochemistry

The sections were stained according to the manufacturer’s protocol. Briefly, the sections were baked at 60 °C for 2 h, dewaxed in turpentine and rehydrated in a graded ethanol series, and then treated with 3% hydrogen peroxide for 10 min to inhibit endogenous peroxidase. The sections were pretreated in a microwave oven (in 0.01 M sodium citrate buffer, pH 6.0) for 5 min, then incubated with p53 antibody (1:100, Santa Cruz) at 4 °C in a humidified chamber overnight. The sections were washed the next day and incubated with the secondary antibody (anti-mouse 1:200, Santa Cruz) for 1 h. HRP-streptavidin (Santa Cruz) was added to the slide. The samples were stained for 5 min with a 0.05% 3, 3′-diaminobenzidine substrate and counterstained with hematoxylin for 5 min and then mounted in neutral balsam. The sections were examined under light microscopy. Expression of p53 in the skin of rats and in patients with arsenic keratosis was analyzed blindly. Positively stained cells were counted and immune intensities of p53 protein in each cell were graded from 0 to 3 (1%–25%, 26%–50%, 51%–75% and 76%–100%, respectively). The sum of all cellular expression levels was calculated to quantify the tissue expression of p53.

### Statistical analysis

Data were analyzed using SPSS 17.0 software (SPSS Inc., Chicago, IL, USA). Student’s *t*-test was performed to analyze differences between groups. The difference was considered to be significant when *P*<0.05.

## Publisher’s Note

Springer Nature remains neutral with regard to jurisdictional claims in published maps and institutional affiliations.
